# A Rare Encounter of Mitral Arcade With Anomalous Papillary Muscles

**DOI:** 10.7759/cureus.21253

**Published:** 2022-01-14

**Authors:** Ranbir Singh, Maureen Wang

**Affiliations:** 1 Internal Medicine, NewYork-Presbyterian Brooklyn Methodist Hospital, Brooklyn, USA; 2 Cardiology, NewYork-Presbyterian Brooklyn Methodist Hospital, Brooklyn , USA

**Keywords:** mitral regurgitation, mitral stenosis, mitral arcade, cardiothoracic surgery, cardiology

## Abstract

A congenital mitral arcade is a rare heart structural abnormality that affects the structure of the papillary muscles and chordae tendinae. This structural deviation impacts the mitral valve’s functional capability, which is why most patients with this condition develop complications such as mitral stenosis or regurgitation. Some patients can obtain successful mitral valve repair. However, most will still need to get a mitral valve replacement. This case presents a young female who was found to have a mitral arcade and underwent mitral valve repair.

## Introduction

Congenital mitral arcade is a rare congenital anomaly of the mitral valve and its tensor apparatus. It is characterized by elongated papillary muscles connected to the tip of the anterior mitral leaflet by a bridge of fibrous tissue [1]. It is thought to result from the arrested embryonic development of the mitral valve before the lengthening and attenuation of the chordae tendineae [2]. Survival into adulthood is rare because of progressive mitral regurgitation or mitral stenosis. We present a case of a 24-year-old female whose transesophageal echocardiogram showed signs of a mitral arcade.

## Case presentation

The patient is a 24-year-old female with a history of palpitations and mitral regurgitation who came to the Brooklyn Methodist Emergency Department with complaints of palpitations. The patient has an extensive 14-year history of intermittent palpitations, lasting for about 30 minutes, triggered by warm showers, coffee, or anxiety, and relieved after the Valsalva maneuver. On the morning of her admission, her palpitations were much worse than usual, lasting for one hour. Valsalva maneuver did not alleviate her palpitations, prompting her to seek immediate medical care. The patient denied a history of rheumatic fever.

**Figure 1:**
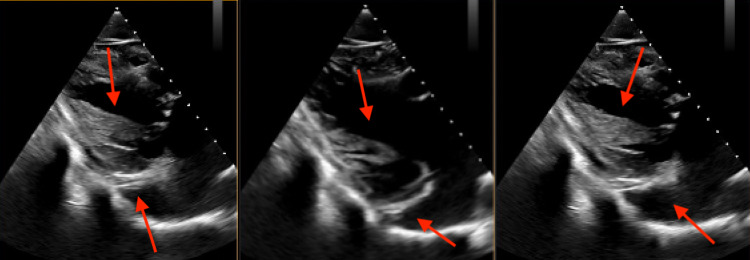
Transesophageal echocardiogram of the patient’s mitral valve Given the doming of the mitral valve, shortened chordae, and restricted papillary muscles as marked by the red arrows, these findings are all consistent withhammock mitral valve (mitral arcade)

While in the emergency room, her EKG showed signs of atrial flutter, which resolved after being placed on Cardizem 60mg Q6H for 24 hours, followed by Cardizem 120 mg daily as her maintenance dose. A transesophageal echocardiogram was obtained, which showed an ejection fraction of 52%, and severe mitral valve regurgitation originating from inadequate coaptation of the anterior and posterior leaflets. After much review, it was determined that the patient’s doming mitral valve, shorted chorda, and restricted papillary were consistent with the hammock mitral valve (Figure 1). On hospital day 10, the patient was in the operating room for complex mitral valve repair with posterior autologous pericardial patch leaflet augmentation, delamination of posterior leaflet and posterior papillary muscle, and mitral annuloplasty. Intra-operative findings were severe posterior leaflet restriction with arcade mitral valve, mild-moderate anterior leaflet restriction, thickened leaflets at the margin, no thickened subvalvar apparatus - papillary muscles inserted almost directly into a posterior leaflet, and some short chordae directly from posterior wall to leaflet.

Post-operation was complicated by acute blood loss anemia of 6.2 mg/dL requiring one unit of packed red blood cells and atrial fibrillation requiring a continuous amiodarone infusion. On hospital day 11, the patient’s hemoglobin improved to 7.4 mg/dL, and atrial fibrillation was ceased. The continuous amiodarone infusion was stopped, and oral amiodarone was started. On hospital day 13, the patient was started on coumadin. On hospital day 14, the patient was discharged with follow-up at the clinic within two weeks. The patient followed up in the clinic on post-op day 35, where the sutures were removed at the sternal incision site. During that visit, the patient denied any new episodes of palpitations. Her EKG showed a heart rate of 86 bpm and sinus rhythm. She left the clinic with plans to follow up with her cardiologist and new primary care physician within one month. 

## Discussion

Hammock mitral valve (or mitral arcade) is an exceedingly rare malformation of the mitral apparatus. It is defined as severe shortening or the complete absence of chordae tendineae [2]. The coupling of shortened chordal apparatus is often seen with anomalies in papillary muscles. The malformation is thought to result from arrest of mitral valve development at a stage after the loss of muscle in chordae and leaflets but before the final attenuation and elongation of mitral chordae have occurred [3]. Anomalous mitral arcade is the most difficult mitral valve to correct because the subvalvular apparatus is difficult to expose, and the considerable amount of muscle underneath the leaflet causes obstruction of the left ventricular inflow, requiring valve replacement [4]. There have only been three cases where patients had a “partial” hammock mitral valve described by Aramendi et al. and Deo et al. as only the posterior leaflet being involved that have undergone successful mitral valve repair over replacement [5,6].

There have been few other case reports on patients presenting mitral arcade. One case report by Fritz et al. discovered a 60-year-old female with a history of severe mitral stenosis admitted for worsening dyspnea. The transesophageal echocardiogram found the mitral arcade and subsequently had a valve replacement [2]. Another case presented by Deepti et al. involved a 13-year-old female with exertional dyspnea and congestive heart failure. The echocardiogram revealed severe mitral stenosis due to anomalous mitral arcade complicated by severe pulmonary hypertension and underwent mitral valve repair [7]. These case reports support the claim that mitral arcades can be found in various age groups. 

This patient has a family history of cardiac abnormalities. Her mother was diagnosed with hypertrophic cardiomyopathy, requiring a heart transplant in her 30s. Although mitral arcade is not fully understood given its rarity, her case supports the idea that direct family history of cardiac diseases increases the risk of developing mitral arcade. Her pathology became complicated by atrial aflutter and a reduced ejection fraction. Her atrial flutter resulted from her dilated left atrium that was caused by her severe mitral regurgitation. Mitral arcade should be considered in the differential diagnosis when a young patient presents with arrhythmia and severe mitral valve regurgitation.

## Conclusions

Mitral arcade is a rare congenital condition that impacts the structure of a patient’s mitral valve. This can lead to complications including mitral stenosis or regurgitation. It can be newly discovered in a broad age range of patients as mentioned in the previous case reports. These patients will often present with dyspnea or palpitations as noted in this case. The ultimate treatment is either a mitral valve repair or replacement. It appears that a direct family history of cardiac disease places patients at risk of developing mitral arcade as the patient’s mother had a history of hypertrophic cardiomyopathy requiring a heart transplant. When assessing a patient with mitral stenosis or mitral regurgitation with a family history of structural cardiac disease, mitral arcade should be considered as part of the differential diagnosis.
